# Trajectories of depression symptom change during and following treatment in adolescents with unipolar major depression

**DOI:** 10.1111/jcpp.13145

**Published:** 2019-10-24

**Authors:** Sian Emma Davies, Sharon A.S. Neufeld, Eleonore van Sprang, Lizanne Schweren, Rogier Keivit, Peter Fonagy, Bernadka Dubicka, Raphael Kelvin, Nick Midgley, Shirley Reynolds, Mary Target, Paul Wilkinson, Anne Laura van Harmelen, Ian Michael Goodyer

**Affiliations:** ^1^ Department of Psychiatry University of Cambridge Cambridge UK; ^2^ Department of Psychiatry Amsterdam UMC VUmc Amsterdam The Netherlands; ^3^ Department of Psychiatry University Medical Center Groningen Groningen The Netherlands; ^4^ MRC Cognition and Brain Sciences Unit University of Cambridge Cambridge UK; ^5^ Research Department of Clinical, Educational and Health Psychology Division of Psychology and Language Sciences University College London London UK; ^6^ Department of Psychiatry University of Manchester Manchester UK; ^7^ School of Psychology and Clinical Language Sciences University of Reading Reading UK

**Keywords:** Depression, therapy, longitudinal studies, outcome

## Abstract

**Objective:**

To classify a cohort of depressed adolescents recruited to the UK IMPACT trial, according to trajectories of symptom change. We examined for predictors and compared the data‐driven categories of patients with a priori operational definitions of treatment response.

**Method:**

Secondary data analysis using growth mixture modelling (GMM). Missing data were imputed. Trajectories of self‐reported depressive symptoms were plotted using scores taken at six nominal time points over 86 weeks from randomisation in all 465 patients.

**Results:**

A piecewise GMM categorised patients into two classes with initially similar and subsequently distinct trajectories. Both groups had a significant decline in depressive symptoms over the first 18 weeks. Eighty‐four per cent (84.1%, *n* = 391) of patients were classed as ‘continued‐improvers’ with symptoms reducing over the duration of the study. A further class of 15.9% (*n* = 74) of patients were termed ‘halted‐improvers’ with higher baseline depression scores, faster early recovery but no further improvement after 18 weeks. Presence of baseline comorbidity somewhat increased membership to the halted‐improvers class (OR = 1.40, CI: 1.00–1.96). By end of study, compared with classes, a clinical remission cut‐off score (≤27) and a symptom reduction score (≥50%) indexing treatment response misclassified 15% and 31% of cases, respectively.

**Conclusions:**

A fast reduction in depressive symptoms in the first few weeks of treatment may not indicate a good prognosis. Halted improvement is only seen after 18 weeks of treatment. Longitudinal modelling may improve the precision of revealing differential responses to treatment. Improvement in depressive symptoms may be somewhat better in the year after treatment than previously considered.

## Background

Adolescence denotes the highest incidence risk rate period for the emergence of major depression (MD) over the lifecourse (Avenevoli, Knight, Kessler, & Merikangas, 2008). The effectiveness of current treatment strategies, both psychological and SSRI medication alone or in combination (NICE, 2015), have moderate effect sizes of between 0.3 and 0.6 (March et al., 2004; Weisz et al., 2017). At least 20% of adolescents with MD show no treatment response (Goodyer et al., 2008) but the reasons for this are unclear. Currently, there are large variations in the definition of response between trials (Berlim & Turecki, 2007). Such discrepancies lower comparability between studies and impact the proportions of patients considered responders or nonresponders, respectively (Uher et al., 2010; Vitiello et al., 2011). Response definitions are based on percentage symptom reduction (treatment response) or final scores below an a priori cut‐off (clinical remission). These methods are arbitrary and may lack clinical meaning (Thibodeau et al., 2015; Uher et al., 2010). Furthermore, there can be an overlap of patients meeting criteria for nonremission (e.g. a final Hamilton Rating Scale for Depression (HRSD) score of ≥7) and a positive clinical response (e.g. reduction of ≥50% in HRSD) (Fu et al., 2008).

Empirical modelling techniques, such as growth mixture modelling (GMM), may address some of the validity issues with a priori definitions, by categorising patients post hoc (Ram & Grimm, 2009). This computational technique searches for naturally occurring heterogeneity to categorise patients into particular latent classes that follow similar trajectories and make no a priori assumptions on what constitutes a meaningful response (Brière, Rohde, Stice, & Morizot, 2016; Thibodeau et al., 2015; Uher et al., 2010). GMM describes the trajectory of relatively homogeneous behavioural groups and how they differ from each other in their shape over time (Gueorguieva, Mallinckrodt, & Krystal, 2011; Ram & Grimm, 2009). Homogeneity of groups also aids investigating predictors of response types that are likely to have small effect sizes.

Growth mixture modelling analyses of treatment trials data in depressed adults reveal a variety of multiple, qualitatively distinct classes, specific predictors and differential therapeutic responses (Cuijpers, van Lier, van Straten, & Donker, 2005; Gueorguieva et al., 2011; Stulz, Thase, Klein, Manber, & Crits‐Christoph, 2010; Thibodeau et al., 2015; Uher et al., 2010). One recent report from the Treatment of Adolescent Depression Study (TADS) noted that, at 12 weeks, there were two groups that improved over the trial, and a further group showing limited change (Scott, Lewis, & Marti, 2019). A depression prevention study noted two groups where symptoms gradually reduced over time; one group showed no change, and the other reported resurgent symptom count within 6 months of the study end (Brière et al., 2016). Unfavourable trajectories have been associated with older age, psychosocial function, higher anxiety symptom levels and psychotic experiences (Gueorguieva et al., 2011; Jeppesen et al., 2015; Perlis et al., 2010; Thibodeau et al., 2015).

The duration of follow‐up plays a contributory role in determining response classes. Thibodeau et al. (2015) found that short‐term follow‐up mistakenly classified some responders as nonresponders, whilst Brière and colleagues (Brière et al., 2016) found a subgroup of adolescents that showed a significant decline in symptoms up to 6 months, but relapsed only after this point. Longer‐term follow‐ups past the end of the treatment may improve the precision of denoting true responders, sustained nonresponders and relapsing patients.

### Objectives

Our primary objective was to reveal trajectories of depression symptoms from randomisation to the final assessment, approximately one year following the end of treatment. The specific aims were to: (a) define the number and shape of longitudinal classes of patients revealed from depression symptoms only; and (b) compare the defined groups with standard a priori definitions of response/remission.

Our second objective was then to test whether selected baseline demographic and clinical characteristics would predict class membership.

## Methods

### Study design

This study was a reanalysis of data from the Improving Mood with Psychoanalytic and Cognitive Therapies (IMPACT) trial (Goodyer et al., 2017a; 2017b). As there we no difference in clinical effects between psychological treatments, these were collapsed for the present study, to investigate whole population. Self‐reported depressive symptoms were measured at six nominal time points: baseline, 6, 12, 36, 52 and 86 weeks postrandomisation. The last two time points were post treatment which was completed by 36 weeks in >95% of the cohort.

### Participants

Adolescents aged between 11 and 17 years, with major depression (DSM‐IV American Psychiatric Association, 2000), were enrolled from 15 UK National Health Service (NHS) clinics, 5 each in North London, North West England and East Anglia. Patients with a lifetime history of mania were excluded. Full details on patient inclusion and exclusion criteria can be found in the study protocol (Goodyer et al., 2011). Four hundred and sixty five patients who were included in the trial had data available for the current analysis (Goodyer et al., 2017a; 2017b).

### Variables

Symptom trajectory class membership was defined using the self‐reported Mood and Feelings Questionnaire (MFQ) score across all time points. This is a 33‐item Questionnaire (Burleson Daviss et al., 2006) of depressive symptomatology covering the past 2 weeks measured on a 3‐point scale (almost never, sometimes, often/almost always). Higher sum scores (range of 0–66) indicated more depressive symptoms and were positively correlated with greater psychosocial impairment (Goodyer et al., 2017a; 2017b).

Baseline variables investigated for their potential predictive value for trajectory class membership were as follows: sum scores from self‐report measures for anxiety (the Revised Children's Manifest Anxiety Scale, RCMAS, Reynolds & Richmond, 1978), obsessionality (the short Leyton Obsessional Inventory for adolescents (LOI), (Bamber, Tamplin, Park, Kyte, & Goodyer, 2002) and psychosocial impairment (the Health of the Nation Outcome Scales for Children and Adolescents, HoNOSCA, Gowers et al., 1999). Lifetime suicide attempts were defined as binary variables (yes, no) from data derived from the Columbia Suicide Severity Rating Scale (Posner et al., 2011). Lifetime nonsuicidal self‐injury was measured using the self‐report Risk and Self Harm Inventory for adolescents (Vrouva, Fonagy, Fearon, & Roussow, 2010). The Kiddie‐Schedule for Affective Disorder and Schizophrenia (K‐SADS, Kaufman et al., 1997) interview assessed psychiatric symptoms and diagnoses. Comorbidity was defined on an ordinal scale, as the number of concurrent diagnoses. Psychotic symptoms were counted from an ordinal scale (absent, present: subthreshold, or present: threshold) obtained from either of the two K‐SADS screening questions for psychosis.

### Statistical methods

#### Imputation

Multiple imputation was used in order to maximise sample size and reduce selection bias. Due to a wealth of auxiliary variables predicting missingness, data were presumed to be missing at random. Additional nonmissing variables were also included to improve model prediction. Full details of imputation rationale and statistical procedure are given in Appendix S1.

#### Growth mixture models

There was substantial variation in the timing of each assessment and to model symptom change accurately, the mean time of each actual assessment (weeks) was taken as the focal point for GMMs. This corresponded to baseline, 12, 18, 43, 60 and 95 weeks postrandomisation. Variation in time of assessment was further included as a covariate in all growth models.

We tested GMMs using the Mplus program version 8.0 (Muthén & Muthén, 2017). Four growth trends were considered: a linear and quadratic growth trend and two linear‐piecewise growth trends. We investigated piecewise growth trends because we hypothesised that different rates of improvement might occur during treatment versus follow‐up stages of the trial. Due to the average length of treatment falling between the means of two assessment time points, we considered two possible transition points in the models: the first was placed at the third assessment (18 weeks on average from baseline) and the second at the fourth assessment (43 weeks from baseline).

Classes were incrementally added to the single class model to determine the best fit. All models allowed for within‐class variation, whereby patient's symptom scores vary around the mean of the group. However, upon testing, there was no evidence of significant variation between classes, (See Appendix S1 and Table 1), meaning that the variation around each group mean was not significantly different for the respective classes. Consequently, between‐class variation was held equal for all growth factors. Only solutions that were replicated with different starting values were accepted. We considered models with 1–5 trajectory classes, and retained the most parsimonious model. Full details of statistical rules for determining parsimony are given in Appendix S1.

**Table 1:** Model fit information for piecewise GMM‐CIs

Fit Statistics	1 Class	2 Classes	3 Classes	4 Classes	5 Classes
GMM‐CIs					
LL (No. of parameters)	−10,487.996	−10,454.836	−10,440.533	−10,431.253	−10,423.269
AIC	21,015.992	20,957.672	20,937.065	20,926.506	20,918.538
BIC	21,098.832	21,057.081	21,053.042	21,059.051	21,067.651
Entropy	1	.844	.734	.718	.729
Group size (%)					
C1	465 (100%)	391 (84.1%)	329 (70.7%)	191 (41.1%)	219 (47.1%)
C2	–	74 (15.9%)	77 (16.6%)	161 (34.6%)	109 (23.5%)
C3	–	–	59 (12.7%)	57 (12.3%)	56 (12.0%)
C4	–	–	–	56 (12.0%)	54 (11.6%)
C5	–	–	–	–	27 (5.8%)

#### Baseline clinical characteristics and predictors of class membership

Univariate analyses were conducted (chi‐square, or t‐tests) to determine whether there were significant differences between classes on baseline demographic and clinical characteristics. Mann–Whitney–Wilcoxon tests were performed where data were non‐normal. Multinomial logistic regression was then used to determine which variables predicted class membership. R‐squared statistic indicated how much variance the regression model explained. Odds Ratios are reported for all predictors.

#### Agreement between categorical definitions and GMM model result

Cohen's Kappa coefficient (Cohen, 1960) was used to test the agreement between trajectory classes and the definitions of: (a) treatment response/nonresponse (50% reduction/or not in MFQ score by end of study) or (b) clinical remission/nonremission (MFQ score at 12 months below or above 27) (Kent, Vostanis, & Feehan, 1997).

## Results

### Participants

All 465 participants who entered the trial were available for the longitudinal analysis. Across all assessments, 65% or more of the sample (304/465) had full data on all MFQ items.

### Outcome data

**Figure 1:**
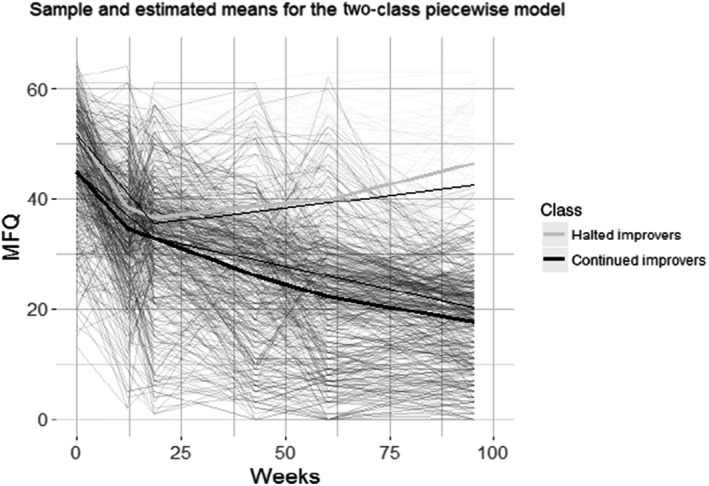
Sample (shown in large grey/black) and estimated (shown in small black) means for the two‐class piecewise growth mixture model. Class 1 reveal a continued‐improver class, *n* = 391 (84.1%) of the population. Class 2 reveal halted‐improver class, *n* = 74 (15.9%) of the population. Behind plots every individual patient's trajectory

Fit information for all piecewise class models tested is provided in Table 1. A two‐class, piecewise model that separately modelled the change in depressive symptoms, linearly, over the first 18 weeks of treatment (on average; assessments 0–2), and then the remaining period of the trial (assessments 3–5), was identified as the optimal model. This is illustrated in Figure 1. BIC showed a favourable decrease of approximately 42 with the addition of a second class from the single class solution. Although the 3‐class solution yielded a slightly lower BIC (ΔBIC = 4.039), the decrease in entropy below commonly accepted thresholds suggests poor classification precision in the 3‐class solution (entropy = 0.844 vs. 0.734). The Mplus code for the two‐class model is provided in Appendix S2.

The two‐class piecewise GMM divided patients into a comparatively large class of 391 (84.1%; class 1) and a small class of 74 (15.9%; class 2). The mean depression scores and the percentage change between time points are shown in Table 2.

**Table 2:** Estimated and observed mean values for MFQ scores and observed mean percentage improvement in MFQ scores are given for both latent classes

Assessment point in average weeks from baseline	Class 1: Continual improvement (*n* = 391)				Class 2: Halted improvement (*n* = 74)			
	MFQ sum scores				MFQ sum scores			
	Estimated	Weighted estimates	Observed	% observed improvement from baseline	Estimated	Weighted estimates	Observed	% observed improvement from baseline
0	44.828	44.810	44.774		51.638	51.721	52.096	
12	37.073	34.649	34.623	22.672	41.090	38.191	38.420	26.252
18	32.881	32.752	32.689	26.991	35.390	36.134	36.558	29.826
43	25.877	23.938	25.920	42.109	39.232	38.243	38.669	25.774
60	23.039	22.291	22.224	50.364	40.789	39.034	39.835	23.535
95	17.247	17.797	17.673	60.528	43.966	44.978	46.357	11.016

Class 2 on average showed significantly higher baseline levels of MFQ scores than class 1 (Wald *X* ^2^(1) = 25.577, *p* < .001). Both classes showed a significant decrease in MFQ score over the first 18 weeks of the trial (−6.466, *p* < .001, and −8.794, *p* < .001, respectively). There was however, a significantly faster rate of MFQ reduction of class 2 compared with class 1 over the first 18 weeks (Wald *X* ^2^(1) = 5.446, *p* = .0196).

For class 2, there was no further statistical difference in rate of change over the rest of the trial (0.899, *p* = .183). Conversely, class 1, on average, continued to show a significant decline in MFQ score, albeit slower than in the initial 18 weeks (−1.639, *p* = .014). This difference between the second linear slopes of the two classes was statistically significant (Wald *X* ^2^(1) = 167.075, *p* < .001). By the end of the trial, Class 1 showed on average a 60.5% improvement in depressive symptoms, compared with 11.0% in class 2. We labelled class 1 as ‘continued‐improvers’ and class 2 as ‘halted‐improvers’.

### Baseline characteristics of each class

The characteristics of patients following each trajectory class are described in Table 3.

**Table 3:** Characteristics of subjects following the two latent trajectories

	Class 1: Continued‐improvers (*n* = 391)		Class 2: Halted‐improvers (*n* = 74)		Comparison	
	Mean (*n*)	*SD* (%)	Mean (*n*)	*SD* (%)	*X* ^2^/*t*/*W*	*p*
Demographics						
Female	285	72.8%	63	85.1%	4.955	.026
Age	15.6	1.4	15.7	1.3	0.459	.647
Region	–	–	–	–	2.035	.361
East Anglia	161	41.2%	24	32.4%	–	–
North London	105	26.9%	22	29.7%	–	–
North West England	125	32.0%	28	37.8%	–	–
Ethnicity (white)	325	83.1%	65	87.8%	1.024	.312
Index of multiple deprivation (IMD)^a^	23.4	–	27.7	–	13,446	.336
Baseline clinical characteristics						
RMAS^b^	40.7	7.3	42.3	6.7	1.863	.065
LOI^c^	9.6	5.1	11.8	5.6	3.124	.002
Suicidal thoughts	345	88.2%	69	93.2%	1.600	.206
Suicidal attempts	131	33.5%	28	37.8%	0.519	.471
NSSI	218	55.8%	53	71.6%	6.443	.011
HONOSCA (available for 435 patients)	18.3	6.0	19.9	6.3	2.018	.046
Comorbidity^d^	–	–	–	–	10.20	.006
1	121	30.9%	26	35.1%	–	–
2	59	15.1%	18	24.3%	–	–
3	5	1.3%	3	4.1%	–	–
4	0	0%	1	1.4%	–	–
Psychotic symptoms	–	–	–	–	2.024	.363
Subthreshold	87	23.6%	16	22.5%	–	–
Threshold	32	8.8%	10	14.1%	–	–
Treatment characteristics						
Treatment arm	–	–	–	–	2.463	.292
BPI	127	32.5%	28	37.8%	–	–
CBT	127	32.5%	27	36.5%	–	–
STPP	137	35.0%	19	25.7%	–	–
Baseline SSRI prescription	87	22.3%	10	13.5%	2.877	.090

a IMD: Median reported for nonparametric tests.

b Revised Manifets Anxiety Scale‐ self‐report.

c Leyton Obsessional Inventory‐ self‐report.

d Due to insufficient cell size, variable was recorded as 0,1 and 2+ to meet assumptions of chi‐square test.

There were more females in the ‘halted’ compared with the ‘continued’ improvers class (85% vs. 73%). No other demographic characteristics were significantly different between groups.

Compared with. ‘continued‐improvers’, ‘halted‐improvers’ on average showed significantly higher obsessionality (LOI) and HoNOSCA scores, more nonsuicidal self‐injury (NSSI), and greater numbers of comorbid diagnoses at baseline. Interestingly neither baseline levels of suicidal attempts, psychotic symptoms nor treatment group discriminated between the classes. There were no significant differences between the two classes on any baseline characteristic (Table 3). Trajectories of classes were similar for HoNOSCA as for MFQ, as a concurrent validation check (Appendix S3).

### Predictors of trajectory class membership

Class 1 acted as the reference class for the logistic regression (Table 4). When controlling for variables included in the model, only the number of comorbid psychiatric diagnoses at baseline significantly predicted a higher probability of membership to halted‐improvers compared with continued‐improvers (Table 4). With each increasing number of comorbid diagnoses the odds of a patient belonging to the ‘halted’ compared with the ‘continued‐improvers’ class increased by a factor of 1.4 (*X* ^2^(4) = 46.03, *p* < .001). The finding however only explained 5.4% of the total variance in class membership (Cox and Snell *R* ^2^ = .054).

**Table 4:** Baseline predictors of trajectory class membership: clinical characteristics

	Class 1: Halted‐improvers	
	OR	95% CI
Gender	0.50	0.23–1.02
Age	1.02	0.84–1.26
RCMAS	0.98	0.94–1.03
LOI	1.06	0.99–1.12
HONOSCA	1.03	0.98–1.08
Attempts	0.98	0.53–1.79
NSSI	1.77	0.95–3.41
Psychotic symptoms	0.97	0.64–1.45
Comorbidity	1.40*	1.00–1.96*

* <.05.

### Agreement between classes and categorical definitions of response

**Figure 2:**
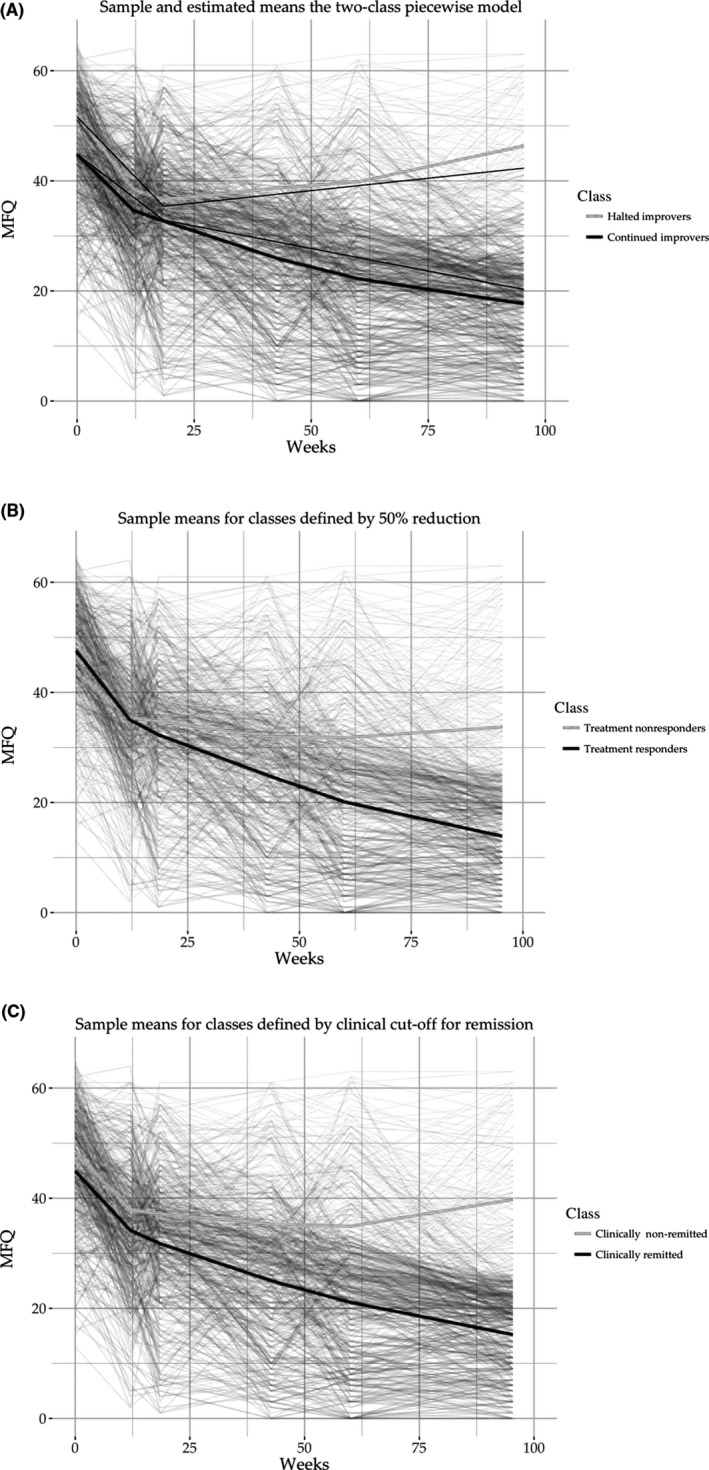
Sample means for the three categorical approaches. (A) Trajectory classes. (B) Percentage reduction for treatment response. (C) Clinical cut‐off for remission. Behind plots every individual patient's trajectory

The agreement in classification between data‐driven classes and a priori categorical classifications are shown in Figure 2.

### Percentage symptom reduction

There was moderate agreement between trajectory class membership and clinical categorical outcome when ‘treatment response’ is defined by percentage reduction of 50% by end of study (*k* = .412, *p* < .001). All halted‐improvers were also treatment nonresponders by this definition. However, only 269 of 391 (69%) of continued‐improvers were also treatment responders. The remaining continued‐improvers (122 of 391; 31%) were classified as treatment nonresponders on the per cent reduction category.

### Clinical cut‐off

There was stronger, albeit still moderate, agreement between trajectory membership and clinical remission, when defined by a cut‐off score of 27 on the MFQ (*k* = .642, *p* < .001) at end of study. All halted‐improvers were also clinical nonremitters. However, only 332 of 391 (85%) of continued‐improvers were classified as in clinical remission. The remaining continued‐improvers (59 of 391; 15%) were classified as not clinically remitted.

Overall, if either a priori category definitions had been used in this study a false negative classification rate of either 15% or 31% would have been reported.

## Discussion

### Key results and interpretation

Using a cohort of depressed adolescents recruited into a clinical trial we computed a depression symptom trajectory that revealed a piecewise function, with two separate linear trajectories. The best fit model was for two classes defined as a large (84.1%) group of continued‐improvers and a small (15.9%) group of halted‐improvers. We noted that both groups improved significantly over the first 18 weeks of the trial. The halted group showed a cessation in improvement from thereon, and this may index a putative relapsing group but this requires replicaton. Although there are studies that support the existence of more than two classes, these applied constraints on investigated trajectory shape (Stulz et al., 2010; Thibodeau et al., 2015) and eliminated within‐class variation from their models (Brière et al., 2016). These methodological choices are often necessary when models struggle to converge (Wickrama et al., 2016). To assume that no individual variation exists within classes in depressive patients, we felt was not representative of real data. We therefore favoured a GMM analysis at outset, which would allow for within‐class variation. Our results were stable across different sets of random starting values without these constraints, offering a much more representative model of patient experience of depressive symptom change.

A striking finding in the present study was the great contrast between trajectories of the two groups across both parts of the model. The faster decline of halted‐improvers may itself be due to the higher depression ratings at baseline. This does not necessarily index ‘good treatment response’: indeed clinicians may need to consider that a fast reduction in depressive symptoms may imply subsequent halted improvement. Further, treatment response cannot be prognostically assessed adequately before approximately 18 weeks. The precise psychological treatment implications however cannot be determined as there was no placebo control group: we do not know whether this period of rapid improvement is due to the psychological therapy, a nonspecific response to receiving assessment and treatment, or regression to the mean. Nevertheless, these findings indicate that clinical care should be taken for those adolescents with greater levels of comorbidity and depression severity at baseline, even when there is an initial rapid response to treatment.

Previous studies reporting two classes describe their trajectory groups as either rapid and gradual responders, or responders and nonresponders (Gueorguieva et al., 2011; Uher et al., 2010). One recent study of adolescents reported three trajectory classes with the two improving classes merging by end of treatment (Scott et al., 2019). However, those studies were limited to short trial durations of 12 weeks or less and were not able to assess longer‐term outcome. The shape of the halted‐improvers trajectory in this clinical study resonates with the symptom resurgence group reported by Briere and colleagues in their community‐sample depression prevention study (Brière et al., 2016). Their resurgence group showed a similar rapid initial improvement in symptoms, followed by a rapid decline in symptoms over time. Whilst the slope value for the second section of our trajectory in the halted‐improvers did not reach significance with this cohort, visual inspection suggests this trend. This trajectory shape suggests that different underlying therapeutic mechanisms may be activated in early and subsequent treatment responses. For example, the halted‐improvers may contain patients who are clinically relapsing as well as not responding. What factors account for the 18‐week assessment as the optimal break point of responding is not clear. Longer‐term follow‐up is essential in future studies to disentangle group trajectory patterns more accurately and to reveal the most valid prognostic markers for treatment response both early and later in follow‐up. Mediation analyses will be required to examine factors and mechanisms involved in early relapse and to separate these from nonresponse.

Despite baseline univariate differences, only comorbidities at baseline was retained as a significant independent predictor with a small odds ratio and low predictive power. We conclude that baseline demographic and clinical observations are insufficient to fully predict depression symptom change over time. We also note that self‐reported illness episode duration was not a moderator of treatment response in the primary results nor here (Goodyer et al., 2008). Although data were not available in this study, we conjecture that, consistent with a number of other randomised controlled trials, a history of childhood maltreatment could have been a moderator of trajectory group membership, with a positive history increasing the liability for being a halted‐improver (Goodyer & Wilkinson, 2019). Further we note that, although patients with a history of mania were excluded from the study, such symptoms may have emerged over the treatment phase. Indeed, we recognise that a next step from this report is to consider what factors over the course of treatment and then follow‐up phases may influence membership of the continued or halted‐improvers groups. We plan to conduct mediation analyses to examine the effects of treatment dose, symptom and psychosocial changes and SSRI prescribing over the treatment phase and their possible impact on membership of continued or halted outcome groups.

Finally, the findings showed only moderate agreement between empirical and a priori definitions of symptom change indexing clinical response or remission. This is similar to results of previous studies (Gueorguieva et al., 2011; Uher et al., 2010). The findings here have provided a clear cut consistent group of halted‐improvers by end of study, potentially predicted by higher depression scores and levels of comorbidity at entry combined with halted improvement by 18 weeks. This suggests caution with the use of cut‐off or percentage change measures as indices of achieving clinical remission, as it is possible that a significant percentage of potentially responding patients might be misclassified too quickly as nonresponders to treatment. Until more is known about subclasses of depression, researchers must take care in their choice of outcome measure and in particular to try to minimise a false negative result.

### Limitations

We cannot use these findings to generalise to population‐based trials where recruitment takes place from schools, community settings or patients with distinct cultural differences to those in this UK NHS study. We do not know if the addition of parent or teacher reporting, as opposed to self‐reported measures would have aided these research findings. GMM are large‐sample statistical techniques, and whilst a sample of 465 is sufficient, a sample of 600 or more may have seen the emergence of a more stable 3‐class model. The lack of nonsymptom driven 86‐week outcome validators, such as interpersonal function, or data on history of childhood maltreatment, are other limitations of this paper. However, investigation of HONOSCA, a measure of functioning as well as psychiatric symptomatology, showed similar trends to MFQ (Appendix S3), which provides preliminary external validity for our findings. In future, GMM trajectories in clinical populations with multiple symptom profile could utilise nondepressive symptoms in their longitudinal analyses.

## Supporting information


**Appendix S1** **.** Statistical methods.
**Appendix S2** **.** Mplus code for the two‐class, piecewise model.
**Appendix S3** **.** Validation with HONOSCA.
