# Supplementary material for: Trajectories of depression symptom change during and following treatment in adolescents with unipolar major depression
Source: J Child Psychol Psychiatry. 2019 Oct 24;61(5):565–74. doi: 10.1111/jcpp.13145 (PMC7216986; doi:10.1111/jcpp.13145)
Supplement: Supplementary file 1 — Appendix S1 . Statistical methods. Appendix S2 . Mplus code for the two‐class, piecewise model. Appendix S3 . Validation with HONOSCA. [file JCPP-61-565-s001.docx]

**Supporting information – Trajectories of depression symptom change during and following treatment in adolescents with unipolar major depression – by Davies *et al*.**

**Appendix S1.** Statistical methods.

*Imputation*

Multiple imputation was favoured over Full Information Maximum Likelihood as auxiliary variables can easily be incorporated into a multiple imputation model and help decrease bias and increase efficiency(Graham, 2003). Variables at all time points were assessed for inclusion in the imputation model in addition to MFQ items. Those related to outcome (p<0.05 or r>=0.3) and/or missingness in outcome, and variables used in final analyses, were included in the model(White, Royston, & Wood, 2011).

This resulted in imputation of 24 variables plus the 33 MFQ items, repeated over six assessments, yielding a dataset too large to impute in wide format. Thus, time-varying data was imputed in long format, a method which is less biased under conditions of less missing data, more repeated measures, and a reliable outcome measure(Gottfredson, Sterba, & Jackson, 2017). For each model, fifty datasets were multiply imputed using chained equations(White et al., 2011). As it is not possible to obtain the VLMR and LMR fit statistics for model comparison in a multiply imputed dataset, multiple imputations were averaged prior to estimation of GMM. While we acknowledge it is more optimal to obtain model estimates from each of the multiply imputed datasets and then combine estimates(White et al., 2011), our approach allowed us to obtain these fit statistics which are crucial for determining the most optimal model.

*Criteria for choosing the most parsimonious trajectory model*

First the model with the lowest AIC and BIC was retained, with BIC values favoured in cases of discrepancy. Models with one fewer latent class were favoured if the BIC difference was 10 or more over the prior model (Uher et al., 2010). Secondly, entropy values were considered. This is a measure of the uncertainty of the model in the classification of subjects into the correct class. Values closer to 1 are preferred. Finally, clinical interpretability and relevance of the class trajectories, as well as class size, were taken into account. Given the sample size of our cohort, models where classes contained less than 10% of the sample were rejected as these were not considered numerically stable(Uher et al., 2010). Patients were assigned to their most likely class based on model probabilities.

*Baseline clinical characteristics and predictors of class membership*

We undertook a two-step approach to investigating trajectories of symptom change, and predictors of these trajectories. After selecting the best fitting trajectory model, we saved the information on most likely class membership for all patients and conducted analyses of associations in a separate step.

*Agreement between classes and categorical definitions of response*

Continued-improvers were considered the comparative for “clinical responders”, and halted-improvers were considered the comparative for “clinical non-responders”. Comparisons are illustrated in Figure 2.

**Appendix S2.** Mplus code for the two-class, piecewise model.

INPUT INSTRUCTIONS

Title: Two_class_GMM

Data: file is 'Mean_Wide_Imputed_MFQ_FINAL.dat';

Variable:

   Names are

        ID gender ageBase arm region time0 randTime adhere famMed imd ethni

        dura0 mfq0 bc0 loi0 rcmas0 rses0 rrs0 rtshR0 rtshSH0 nssi0 attem0

        tho0 sleep0 BehavD0 ComorD0 AnxD0 AnxOth0 MDD0 ssri0 time6 dura6 mfq6

        bc6 loi6 rcmas6 rses6 rrs6 rtshR6 rtshSH6 nssi6 attem6 tho6 sleep6

        BehavD6 ComorD6 AnxD6 AnxOth6 MDD6 ssri6 time12 dura12 mfq12 bc12

        loi12 rcmas12 rses12 rrs12 rtshR12 rtshSH12 nssi12 attem12 tho12 sleep12

        BehavD12 ComorD12 AnxD12 AnxOth12 MDD12 ssri12 time36 dura36 mfq36

        bc36 loi36 rcmas36 rses36 rrs36 rtshR36 rtshSH36 nssi36 attem36 tho36

        sleep36 BehavD36 ComorD36 AnxD36 AnxOth36 MDD36 ssri36 time52 dura52

        mfq52 bc52 loi52 rcmas52 rses52 rrs52 rtshR52 rtshSH52 nssi52 attem52

        tho52 sleep52 BehavD52 ComorD52 AnxD52 AnxOth52 MDD52 ssri52 time86

        dura86 mfq86 bc86 loi86 rcmas86 rses86 rrs86 rtshR86 rtshSH86 nssi86

        attem86 tho86 sleep86 BehavD86 ComorD86 AnxD86 AnxOth86 MDD86 ssri86

        lgTime6 lgTime12 lgTime36 lgTime52 lgTime86;

USEVAR= mfq0 mfq6 mfq12 mfq36 mfq52

             mfq86 time6 time12 time36

             time52 time86;

IDVARIABLE= ID

Class=c(2);

Analysis:

      Estimator = MLR;

     Processors=4;

     STARTS=5000 100;

     TYPE=MIXTURE RANDOM;

Model:

 %Overall%

  i s1 | mfq0@0 mfq6@1.2 mfq12@1.8 mfq36@1.8 mfq52@1.8 mfq86@1.8;

             i s2 | mfq0@0 mfq6@0 mfq12@0 mfq36@4.3 mfq52@6 mfq86@9.5;

     i WITH s1;

     i WITH s2;

     s1 WITH s2;

     mfq6 ON time6;

     mfq12 ON time12;

     mfq36 ON time36;

     mfq52 ON time52;

     mfq86 ON time86;

Output:

     sampstat;

     Tech7;

 PLOT:

     TYPE=PLOT3;

     SERIES = mfq0(0) mfq6(1.2) mfq12(1.8)

     mfq36(4.3) mfq52(6) mfq86(9.5);

 SAVEDATA:

 File="C:\Users\sed48\GMM_CI_2_cprob.csv";

 SAVE=CPROB;

**Appendix S3.** Validation with HONOSCA.

To provide support for the validity of the two trajectory classes, we examined HoNOSCA total scores. HoNOSCA is a putative measure of well-being that includes assessment of symptoms of multiple disorders and functioning. We would expect similarity in trajectory shape, resulting in significant differences between classes at baseline and final time point and an insignificant HoNOSCA scores at the breaking point of 18 weeks. In addition, we compared the percentage decline in HoNOSCA score at their final assessment.

Figure 1 plots the mean trajectory of HoNOSCA scores across trajectory classes. Visual inspection suggests general agreement in depressive symptom trajectories and mean scores of impairment across the two classes.  As expected, class 2 showed a significantly higher baseline HoNOSCA score (19.9), than class 1 (18.3, *t*= -2.02, *p*=.047). In addition, class 2 showed a significantly higher HoNOSCA score at the end of the trial (14.7) compared with class 1 (6.5, t=-8.15, p<.001). Following the pattern shown in the MFQ trajectories, the difference between HoNOSCA scores at the third assessment was insignificant (class 2: 14.3, class 1: 12.8; *t*=-1.25, *p*=.215). Class 2 showed a 26% improvement in HoNOSCA score by the end of the trial, whereas class 1 experienced a 64% improvement. Overall, HoNOSCA scores appear to follow similar trajectories to MFQ. Furthermore, linear regression showed that class membership significantly predicted HoNOSCA score at 86 weeks (Table 2). Class 2 on average have a higher HoNOSCA score by 0.5 standard deviations at 86 weeks than class 1. Together, this offers support that the class model has a level of predictive validity and are measuring clinically relevant changes.


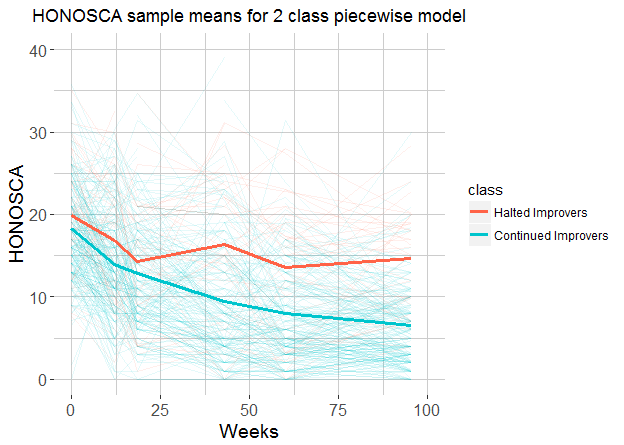


Figure 1: HoNOSCA sample means for the 2-class piecewise growth mixture model, derived from MFQ.  Behind plots every individual patient’s trajectory, colour coded to their respective classes.

Table 2: Regression results for class membership on HoNOSCA scores at 86 weeks.

|  | **R2** | **B** | **SE B** | **β** | **P** |
| --- | --- | --- | --- | --- | --- |
| **Class2** | 0.249 | 8.180 | 0.861 | 0.499 | <.001 |

**References**

Gottfredson, N. C., Sterba, S. K., & Jackson, K. M. (2017). Explicating the Conditions Under Which Multilevel Multiple Imputation Mitigates Bias Resulting from Random Coefficient-Dependent Missing Longitudinal Data. *Prevention Science*, *18*(1), 12–19. http://doi.org/10.1007/s11121-016-0735-3

Graham, J. W. (2003). Adding Missing-Data-Relevant Variables to FIMLBased Structural Equation Models. *Structural Equation Modeling*, *10*(1), 80–100.

White, I. R., Royston, P., & Wood, A. M. (2011). Multiple imputation using chained equations: Issues and guidance for practice. *Statistics in Medicine*, *30*(4), 377–399. http://doi.org/10.1002/sim.4067
